# The prognostic value of cortical stimulation induced seizures using stereo EEG in presurgical evaluation of focal epilepsies

**DOI:** 10.1038/s41598-025-92241-z

**Published:** 2025-03-07

**Authors:** Caroline Reindl, Michaela Unger, Katrin Walther, Johannes D. Lang, Stephanie Gollwitzer, Jeanne Cuny, Stritzelberger Jenny, Tamara M. Welte, Dominique C. Marterstock, Arnd Doerfler, Daniel Delev, Karl Rössler, Sebastian Brandner, Stefan Rampp, Stefan Schwab, Piotr Lewczuk, Hajo M. Hamer

**Affiliations:** 1 Epilepsy Center Department of Neurology, University Hospital Erlangen, Erlangen, Germany; 2 Department of Neurology, University Hospital Erlangen, Schwabachanlage 6, 91054 Erlangen, Germany; 3 Department of Neurosurgery, University Hospital Erlangen, Erlangen, Germany; 4 Department of Neuroradiology, University Hospital Erlangen, Erlangen, Germany; 5 Department of Neurosurgery, University Hospital Vienna (AKH), Vienna, Austria; 6 Department of Neurosurgery, University Hospital Halle (Saale), Halle, Germany; 7 Department of Neurosurgery, Hospital Fürth, Fürth, Germany; 8 Department of Psychiatry and Psychotherapy, University Hospital Erlangen, Erlangen, Germany; 9 Department of Neurodegeneration Diagnostics, Medical University of BiałYstok, Białystok, Poland; 10 Department of Biochemical Diagnostics, University Hospital of BiałYstok, Białystok, Poland

**Keywords:** Stereo-encephalography, Direct electrical stimulation, Seizure, Epileptogenic zone, Seizure pattern, Epilepsy surgery, Surgical outcome, Neurology, Neurological disorders, Epilepsy

## Abstract

The value of stimulation-induced seizures for multimodal determination of the epileptogenic zone in preoperative epilepsy diagnostics has not yet been sufficiently investigated. Patients with focal pharmacorefractory epilepsy who underwent invasive electroencephalography with cortical 50 Hz stimulation at the Epilepsy Center Erlangen between 2018 and 2023, had at least one stimulation-induced seizure, underwent resective epilepsy surgery, and had a postoperative follow-up ≥ 1 year were analyzed. 20 patients were included, 11 (55.0%) with temporal, 7 (35.0%) with frontal and 2 (10.0%) with parietal lobe epilepsy. 12 patients (60.0%) had a good Engel outcome (Engel 1A). Associated with a good vs. poor (Engel 1B-4) surgical outcome were not only the percentage of resected electrode contacts of the spontaneous seizure onset zone, SOZ (*p* = 0.005), but also the stimulation SOZ (*p* = 0.022), as well as stimulation-induced seizure with a typical seizure semiology (*p* = 0.033), the electrodes inducing a stimulation-induced seizure (*p* = 0.014), electrodes with an identical seizure onset pattern (*p* = 0.035), and the occurrence of low voltage fast seizure onset pattern, LVFA (*p* = 0.015). ROC analyses showed that the AUC for the predictors of the spontaneous SOZ were greatest for the stimulation SOZ (AUC 0.876) and stimulation-induced seizures with LVFA (0.860). Analysis of combined predictors showed higher odds of predicting SOZ for combinations including LVFA. Electroclinical stimulation seizures have prognostic value in determining the epileptogenic zone. Characteristics such as the seizure onset zone, seizure pattern and stimulation seizure semiology predict seizure freedom in case of resection of electrode contacts. Electrodes should be resected where both stimulation seizures have been induced or the seizure pattern has been localized and low voltage fast seizure pattern has occurred.

**Supplementary Information:**

The online version contains supplementary material available at 10.1038/s41598-025-92241-z.

## Introduction

For a subset of patients with pharmacoresistant focal epilepsy, resective epilepsy surgery offers the best chance of seizure freedom^1,2^, ranging from 40–80%^3^. The identification of reliable preoperative predictors is therefore essential to plan resection and maximize the chances of postoperative seizure control. This however is challenging as no diagnostic method can preoperatively define the entire epileptogenic zone with certainty^4^. In patients undergoing invasive video-EEG monitoring, cortical stimulation is used for functional mapping of the eloquent cortex and identification of the seizure onset zone (SOZ)^5,6^. The clinical value of stimulation-induced seizures for delineating the epileptogenic zone is still not well established, as it has not been systematically studied in detail. Previous studies have found variable concordance rates of stimulation seizures and spontaneous seizures^7–10^.

High false-positive rates were found especially for extratemporal stimulation^7^. The concordance rate with spontaneous seizures was reported to be higher for electrically induced seizures than for chemically induced seizures^8^. An investigation of stimulation frequency showed that, on the one hand, 1 Hz stimulation may have a specific role in seizure induction, as more typical electroclinical seizures occurred in the sample. However, 1 Hz stimulation seizures were rare outside the medial temporal and insular regions. On the other hand, the 50 Hz stimulation frequency induced a significantly greater number of stimulation seizures overall, with a distribution across all stimulated areas^11^.

Few studies have examined the relationship between seizures that occur during cortical electrical stimulation and seizure outcome after surgery. Oderiz et al. analyzed the prediction to surgical outcome by the results of cortical stimulation in a retrospective study^12^. Their data showed that the proportion of patients with stimulation-induced electroclinical seizures was significantly higher in the group with good outcome compared to the group with poor outcome (71% vs. 48%, *p* = 0.02).

However, electroclinical features of stimulation-induced seizures (SIS) that are of essential localising value for the SOZ and predictive of surgical outcome when the area is removed have not been thoroughly investigated. In the present study, we focussed on 50 Hz stimulation. The aim of this study was firstly to investigate whether the resection of the SOZ identified by cortical stimulation during stereo-EEG is associated with a good surgical outcome and which electroclinical characteristics of SIS are associated with a good surgical outcome. Secondly, electroclinical characteristics of SIS were investigated as predictors of the spontaneous SOZ.

## Methods

### Study cohort

A retrospective analysis was conducted on intracranial EEG recordings of 20 patients with drug-refractory focal epilepsy who underwent invasive EEG (iEEG) monitoring with stereotactically implanted depth electrodes at the Erlangen Epilepsy Center between 2017 and 2023. In order to be included in the study, patients had to meet the following criteria:

(1) iEEG investigation with the recording of at least one spontaneous electroclinical seizure, (2) 50 Hz cortical electrical stimulation during the invasive EEG investigation, (3) at least one stimulation-induced electroclinical seizure (SIS) occurred during stimulation, (4) epilepsy surgery and a minimum postoperative follow-up period of at least one year, (5) availability of a high-resolution 3D data set of T1-weighted MRI images preoperatively, peri-implantation and post-operatively for determination of the resection volume. All patients who met the inclusion criteria were included.

The implantation scheme of the depth electrodes was exclusively based on the putative localisation of the epileptic zone, derived from presurgical non-invasive diagnostics including seizure semiology, non-invasive video-EEG monitoring, 3-T magnetic resonance imaging (MRI) according to a standardised epilepsy protocol, structured neuropsychological examination, and if found appropriate, voxel-based morphometry (VBM), ictal single photon emission computed tomography (SPECT), positron emission tomography (PET) or magnetoencephalography (MEG). Postoperative seizure outcome assessed at 1-year follow-up was classified into seizure free, good Engel outcome (Engel 1A) and poor Engel outcome with remaining seizures (Engel 1B to 4).

All patients gave written informed consent for using their clinical data for scientific studies. All analyses were performed in accordance with local guidelines and regulations. The study was approved by the Ethic committee by the Medical faculty of the Friedrich Alexander University Erlangen (IRB-No 65_20B).

### Intracranial EEG and cortical stimulation

EEG data were recorded with a sampling rate of 2048 Hz (Micromed^®^, Italy). For electrical stimulation a series of electrical pulses of increasing intensity were applied via pairs of adjacent electrodes using biphasic pulses of 0.3 ms at a frequency of 50 Hz, each train lasting for up to 5 s. The stimulation intensity was increased from 1 mA in steps of 2 mA until the occurrence of negative or positive clinical signs, the occurrence of a seizure, the occurrence of local discomfort, or until a maximal intensity of 15 mA was reached. Invasive EEG was displayed with a high pass filter of 0.3 Hz, a low pass filter of 1000 Hz, a gain of 400–1500 µV/cm and a notch filter.

During the intracranial EEG recording, antiseizure medication was withheld in order to record seizures. At the end of the recording and at least 12 h after reinstitution of antiseizure medication, cortical electrical stimulation was performed for the purpose of functional mapping.

Asymptomatic runs of epileptiform discharges or ictal EEG patterns induced by electrical stimulation (after-discharges) were not analyzed in this study^13^. SIS were analyzed with respect to localization of the stimulation electrodes, the type and localization of the seizure pattern and seizure semiology. Semiology was classified as identical if the semiology of spontaneous and induced seizures were the same.

### EEG analysis of seizure pattern

All electroclinical seizure patterns were analyzed independently by two board-certified electroencephalographers (CR, HMH). Cases with disagreement on categorization were discussed between the two electroencephalographers until consensus was reached.

Seizure patterns were classified according to the occurrence of LVFA (low voltage fast activity) and according to the classification described by Lagarde et al.^14^ We distinguished seizure patterns with LVFA: “LVFA alone”, “preictal spiking followed by LVFA”, “slow wave followed by LVFA”, “poly-spikes followed by LVFA”, versus seizure patterns without LVFA: “rhythmic spikes of low frequency”, “sharp theta to alpha activity”, “sharp beta activity” and “delta plus fast activity” (Figure, Supplementary Information).

### Imaging and co-registration of electrode contacts and resection cavities

Co-registration was performed to accurately localise individual electrode contacts anatomically and within the resection cavity. For a detailed description of the methods, we also refer to our previous studies^15,16^. In brief, magnetic resonance imaging was performed according to a standard epilepsy protocol of the Epilepsy Center Erlangen (3 Tesla, Trio Magnetom Sonata, Siemens Healthcare, Erlangen, Germany) for all patients of the study. T1-weighted 3D datasets with a resolution of 1 × 1 × 1 mm were used for further analysis. Algorithms of SPM 12 (https://www.fil.ion.ucl.ac.uk/spm) were used. First, pre- and postoperative T1 sequences were aligned in reference to the anterior and posterior commissure and co-registered in SPM. Second, T1 images were resliced to a 2 × 2 × 2 mm resolution for purposes of manual lesion segmentation. Third, lesions were manually plotted onto the 3D T1 images using MRIcron software (https://www.mricro.com)^17^ and adjusted for a postresection-shift using the preoperative T1 sequence. For this purpose, the lesion delineations were additionally compared with the preoperative 3D T1 images and a manual correction was made for displacement of unresected tissue into the resection cavity. Fourth, lesion maps and postoperative T1 images were normalized with Clinical Toolbox in SPM 12 (https://www.nitrc.org/projects/clinicaltbx/) using enantiomorphic lesion masking and unified normalization-segmentation routines^18,19^ and resulting in normalized images with a resolution of 1 × 1 × 1 mm. The normalized T1 images were used to create an average T1 template and a binary mask for statistical analysis using FSL (https://git.fmrib.ox.ac.uk/fsl/); Jenkins 2012)^20^. Statistical analyses within the binarized brain mask were performed using these normalised lesion maps with a resolution of 1 × 1 × 1 mm. Using an in-house Matlab script (https://www.mathworks.com), we calculated the resected electrodes by the difference of the MNI coordinates of the electrode positions and the normalised lesion. We used MNI152 standard-space T1-weighted average structural template image for 3D-visualization in MRIcroGL (https://www.nitrc.org/projects/mricrogl) and Brainstorm (http://neuroimage.usc.edu/brainstorm)^17,21^.

### Statistical analysis

Statistics of univariate analyses were performed using R Statistics 2023.06 (https://www.R-project.org/)^22^. Proportions were compared using Pearson’s Chi Squared test, continuous variables were compared using the Mann–Whitney U test. Two-sided p-values of less than 0.05 were considered statistically significant. Lesion mapping was computed with NiiStat (https://www.nitrc.org/projects/niistat).

The setting of this study, with multiple electrodes implemented in a patient’s brain (82–133 observations per patient) leads to a hierarchical structure of the dataset with the electrodes clustered within patients. Hence, to model the binary outcome variable, SOZ, as functions of the explanatory variables, Generalized Linear Mixed Models (GLMM) of the binomial family with the logit link function were applied with random intercept accommodating for the intra-cluster homogeneity. In order to evaluate the predictive accuracies of the explanatory variables, separate models were fitted with the predictors of interest, one by a model, and the covariates. The predictive accuracies were post-estimated by evaluation of the Areas Under the Curves (AUC) of the Receiver Operating Characteristics (ROC) following from the models, as detailed by Cleves et al.^23^. The modeling was performed (i) in the total cohort (*n* = 20), (ii) in the subgroup of patients with a postoperative Engel outcome of 1A (*n* = 12) and (iii) in the subgroup of patients with a worse postoperative Engel outcome (≥ 1B, *n* = 8).

Finally, the “full” model was fit with the five explanatory variables characterizing with the best predictive accuracies and the confounders: good Engel outcome, lesional epilepsy, temporal lobe epilepsy, left hemispheric epilepsy, epilepsy onset, duration of epilepsy, female. Based on that model, odds ratios were computed for eight clinically most relevant combinations of pairs of the predictors. All models were fit with Stata 17 (StataCorp, College Station, TX, USA). For the hypotheses testing, *p* < 0.05 was considered significant.

## Results

### Electroclinical characteristics of the cohort

**Fig. 1:**
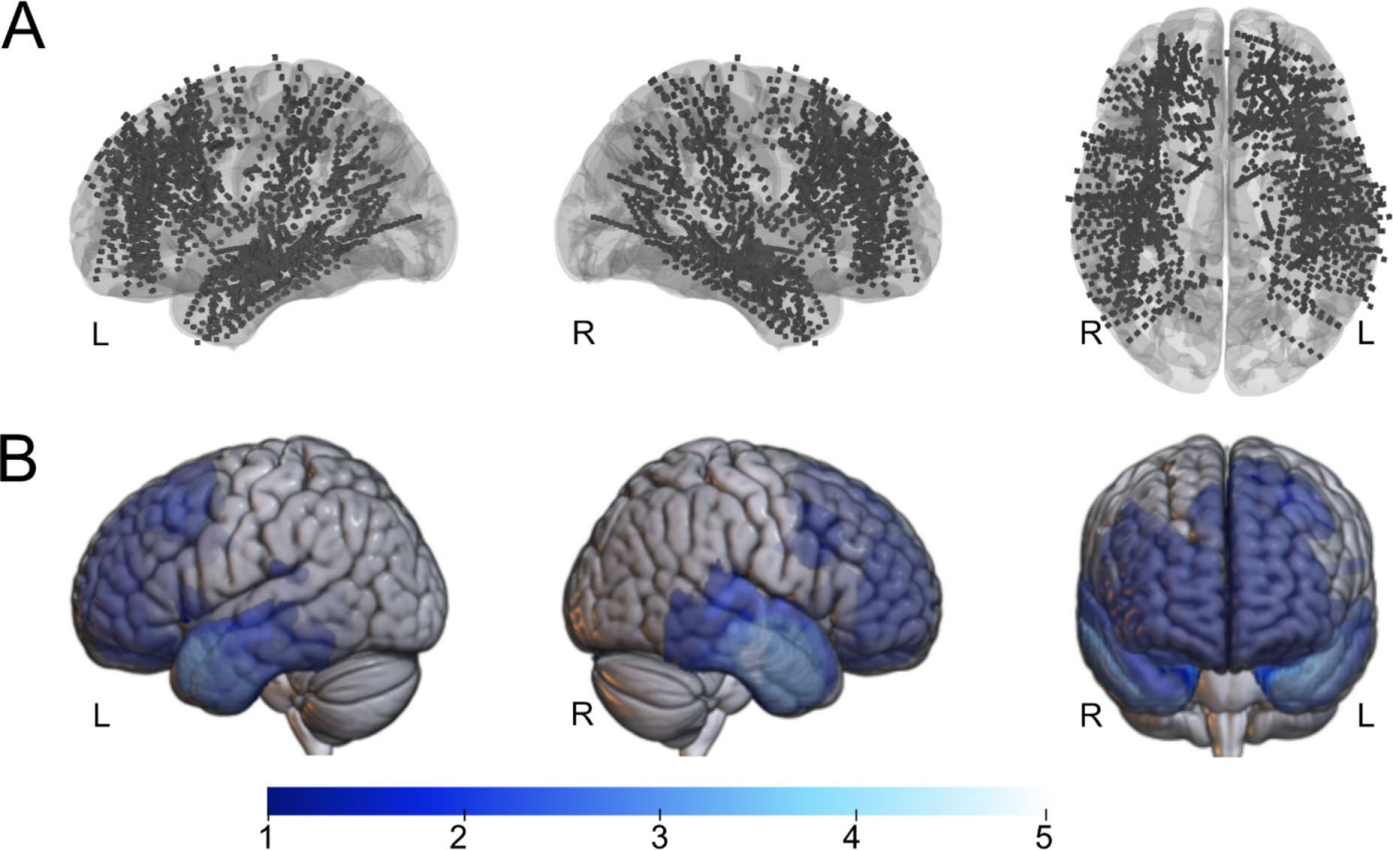
Electrode contacts of total cohort (**A**) and lesion overlap map of total patient group (**B**), results are presented in MNI space, co-registered on a standard MNI152 template. Color bar visualizes number of patients with overlapping resection zones. *R* right, *L* left.

20 patients who underwent invasive EEG investigation in the period from 2017 to 2023 at the Epilepsy Center Erlangen, including cortical stimulation and consecutive epilepsy surgery, with a minimum postoperative follow-up of 1 year, were analysed. 12 (60.0%) were female and the mean (SD) age at the invasive EEG investigation was 34 (11) years. Of the total of 2079 depth electrode contacts in the overall cohort, 813 electrode contacts were localised in the frontal lobe, 743 in the temporal lobe, 106 in the insular region, 187 in the limbic region, 207 in the parietal lobe and 23 in the occipital lobe, Fig. 1. 12 patients (60.0%) had a good Engel-outcome (Engel 1A). We analysed a cohort with different types of focal epilepsy; neither demographic data nor epilepsy type or aetiologies showed significant differences in univariate analysis with regard to postoperative Engel-outcome, Table 1.

**Table 1:** Electroclinical characteristics of patients.

	Total cohort (*n* = 20)	Engel 1A (*n* = 12)	Engel ≥ 1B (*n* = 8)	*p*-value
Demographics				
Female	12 (60.0)	7 (58.3)	5 (62.5)	0.852
Age at epilepsy onset (years)	10 (6–25)	8 (5–14)	21 (7–29)	0.074
Duration of epilepsy (years)	20 (10–27)	22 (16–27)	14 (6–27)	0.373
Age at invasive EEG (years)	33 (27–43)	30 (23–41)	35 (30–49)	0.189
Type of epilepsy				
Temporal lobe epilepsy	11 (55.0)	6 (50.0)	5 (62.5)	0.592
Mesial	4 (20.0)	3 (25.0)	1 (12.5)	0.505
Neocortical	7 (35.0)	3 (25.0)	4 (50.0)	0.263
Extratemporal epilepsy	9 (45.0)	6 (50.0)	3 (37.5)	0.927
Frontal	7 (35.0)	4 (33.3)	3 (37.5)	0.852
Parietal	2 (10.0)	2 (16.7)	0	0.235
Left hemispheric epilepsy	12 (60.0)	7 (58.3)	5 (62.5)	0.852
Etiology of epilepsy				
Lesional				
HS	2 (10.0)	1 (8.3)	1 (12.5)	0.767
Low grade tumor	2 (10.0)	2 (16.7)	0	0.236
FCD type II	8 (40.0)	6 (50.0)	2 (25.0)	0.276
MCD	3 (15.0)	1 (8.3)	2 (25.0)	0.319
Non-lesional	6 (30.0)	3 (25.0)	3 (37.5)	0.560
Invasive EEG				
No. of iEEG electrodes	103 (91–109)	102 (88–113)	104 (85–112)	0.787
No. of SIS per patient	2 (2–3)	2 (2–3)	3 (1–5)	0.713
Seizure onset pattern				
Spont. seizure with LVFA	15 (75.0)	9 (75.0)	6 (75.0)	1.000
SIS with LVFA	10 (50.0)	8 (66.7)	2 (25.0)	0.075
Epilepsy surgery				
Resection volume (ml)	39.2 (12.4–54.2)	46.3 (12.4–60.1)	28.5 (13.9–40.1)	0.135
Percentage of resected electrodes				
Spontaneous SOZ	88.1 (59.4–100.0)	100.0 (86.9–100.0)	50.0 (0–76.2)	**0.005**
SOZ of SIS	39.6 (0–77.1)	70.8 (37.5–89.6)	0 (0–36.4)	**0.022**
SIS inducing electrodes	46.9 (0–85.0)	58.3 (45.0–100.0)	0 (0–38.5)	**0.014**
Identical semiology of SIS	0 (0–47.9)	37.5 (0–81.3)	0 (0–0)	**0.033**
Identical onset pattern of SIS	20.0 (0–75.5)	60.7 (0–100.0)	0 (0–20.0)	**0.035**
LVFA at onset of SIS	0 (0–90.6)	77.1 (0–100.0)	0 (0–0)	**0.015**

Values are n (%) or median (interquartile range).

*N* number, *HS* hippocampal sclerosis, *FCD* focal cortical dysplasia, *MCD* malformations of cortical development, *iEEG* invasive Electroencephalography, *SIS* stimulation-induced seizure, *LVFA* low voltage fast activity, *SOZ* seizure onset zone.

Statistically significant values (*p* < 0.05) are expressed in bold.

### Stimulation-induced seizures (SIS)

In total, we documented 51 SIS, with a mean (SD) of 2.6 (1.3) SIS per patient. 28 (54.9%) of these SIS occurred in patients with good postoperative Engel outcome (Engel 1A). Classification of stimulation induced seizure onset patterns showed 25 (49.0%) seizures with “rhythmic spikes of low frequency”, 8 (15.7%) seizures with “sharp theta to alpha activity”, 2 (3.9%) seizures with “delta-brush”, 14 (27.5%) seizures with “LVFA”, 1 (2.0%) seizures with “preictal spiking followed by LVFA” and 1 (2.0%) seizures with “slow wave followed by LVFA”.

### Stimulation-induced seizures (SIS) and surgical outcome

**Fig. 2:**
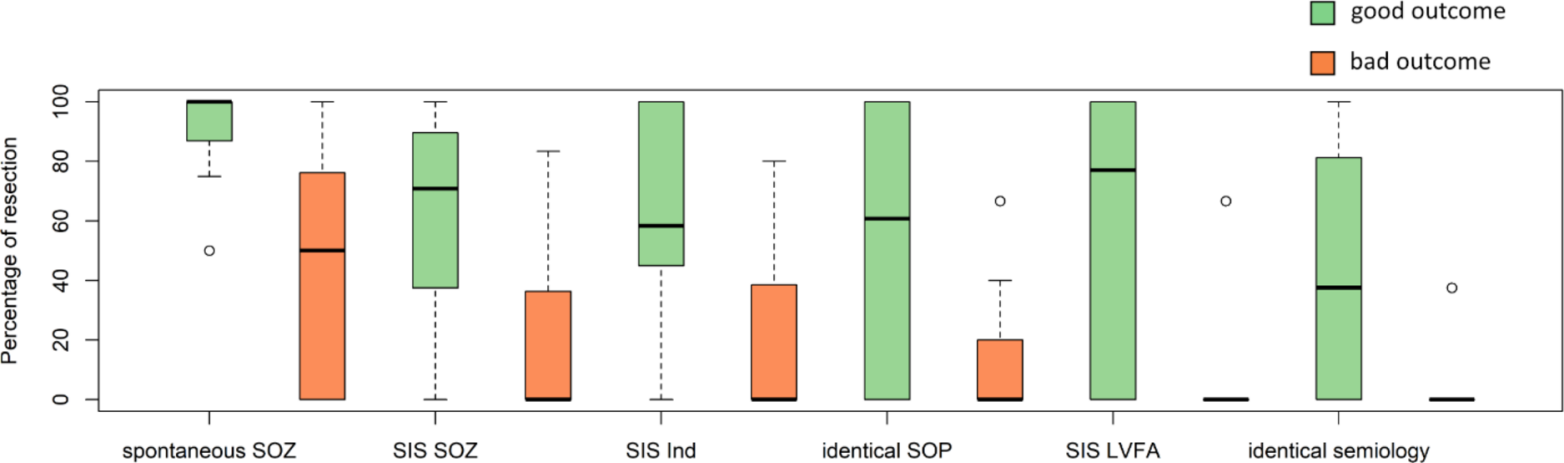
Percentages of resected electrode contacts in patients with good or poor postsurgical outcome. *SOZ* seizure onset zone, *SIS* stimulation-induced seizure, *LVFA* low voltage fast activity, *SOP* seizure onset pattern, *Ind* inducing electrode contacts, good outcome (Engel 1A), bad outcome (Engel ≥ 1B).

Univariate analyses revealed that the percentage of resected cortical stimulation–induced SOZ electrode contacts was higher in the good than in the poor outcome group (70.8% [37.5–89.6] vs. 0% [0–36.4]; *p* = 0.022). A higher number of electrodes were resected in the good outcome group as opposed to the poor outcome group for the SIS inducing electrode contacts (58.3% [45.0–100.0] vs. 0% [0–38.5]; *p* = 0.014). The percentage of resected electrode contacts was higher in the good than in the poor outcome group for electrodes involved in the SOZ with an identical semiology of SIS (37.5% [0–81.3] vs. 0% [0–0]; *p* = 0.033), the electrodes involved in the SOZ of SIS with an identical onset pattern (60.7% [0–100.0] vs. 0% [0–20.0]; *p* = 0.035) and electrodes with LVFA at seizure onset of SIS (77.1% [0–100.0] vs. 0 [0–0]; *p* = 0.015), Table 1; Fig. 2. The median (range) percentage of resected electrodes of spontaneous SOZ revealed similar results and was higher in the good than in the poor outcome group (100% [85–100] vs. 50% [0–81]; *p* = 0.005). The stimulation intensity leading to SIS did not differ significantly between patients with good vs. poor postoperative outcome: 8.5 (4.25–10.0) vs. 7.0 (5.58–11.3); *p* = 0.786.

### Stimulation-induced seizures (SIS) as the predictors of the spontaneous seizure onset zone

**Fig. 3:**
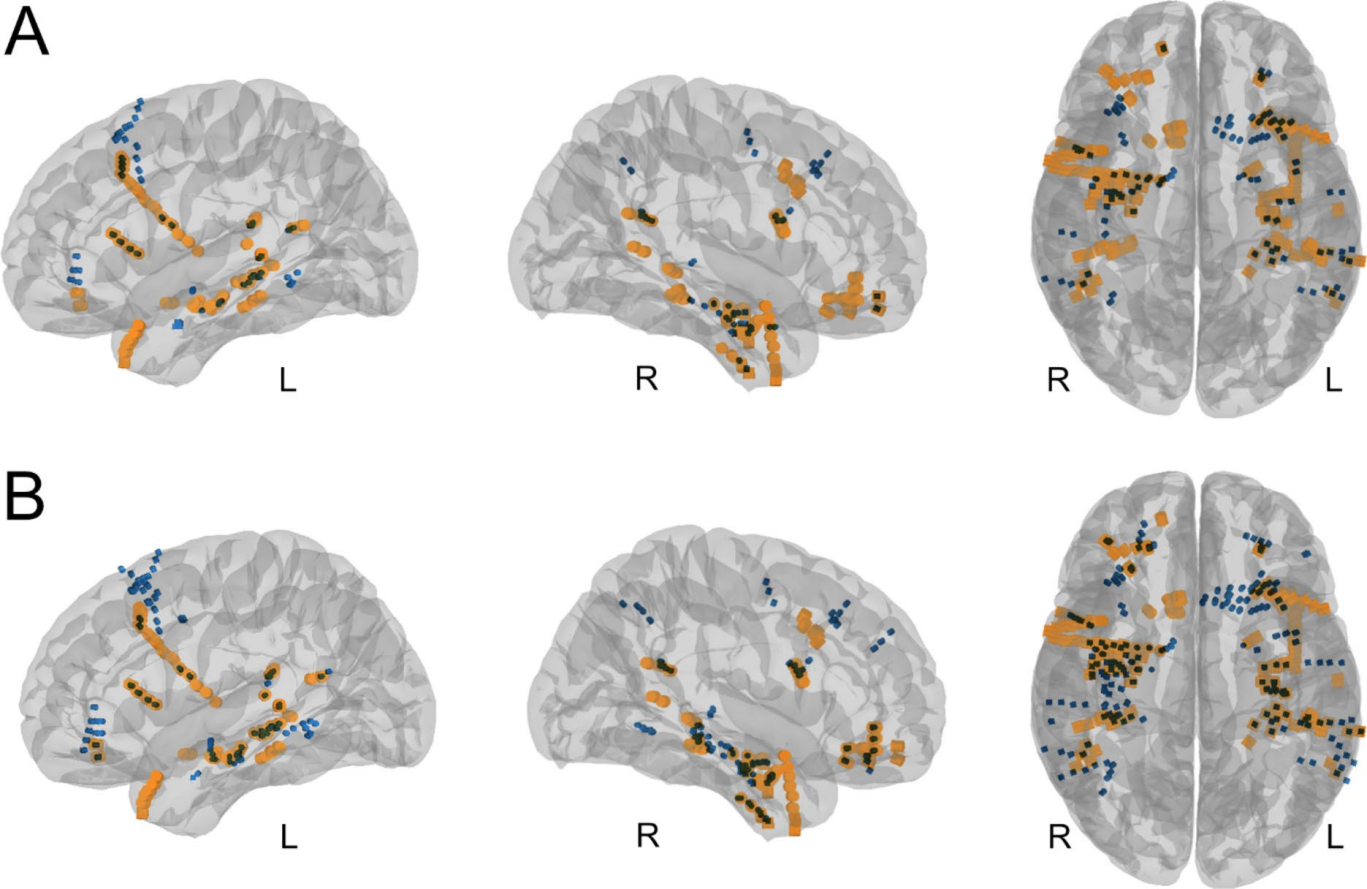
Electrode contacts in MNI space, co-registered on a standard MNI152 template (**A**) Electrode contacts inducing stimulation-induced seizures are depicted in blue. (**B**) Electrode contacts involved in stimulation seizure onset are depicted in blue. For **A + B** electrode contacts associated with spontaneous seizure onset are color-coded orange. The overlap of orange with blue-colored electrodes indicates concordant localization of the stimulation-induced seizure onset zone and the spontaneous seizure onset zone. *R* right, *L* left.

**Fig. 4:**
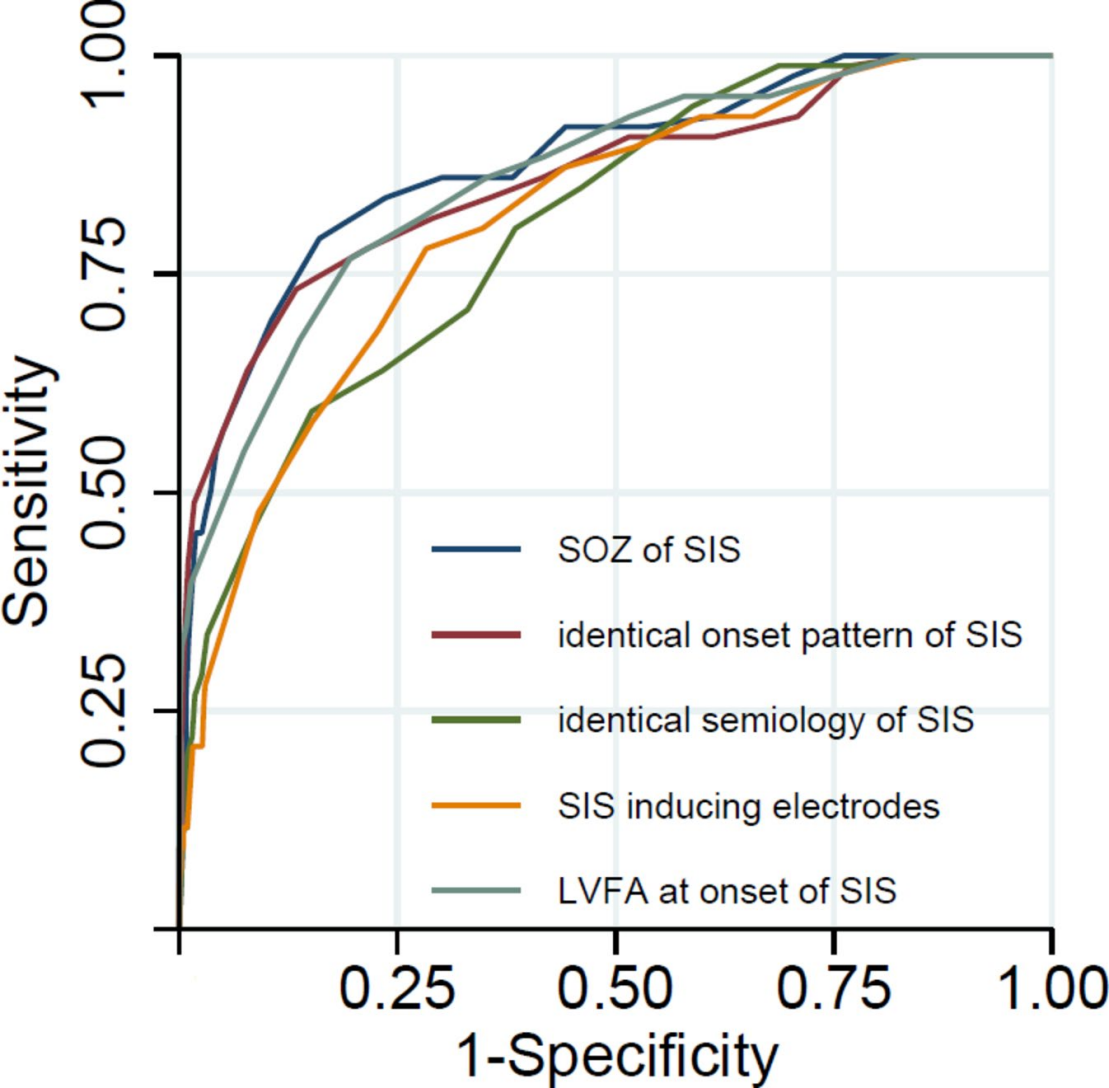
Receiver operating characteristic curves resulting from the models in the subgroup of patients with Engel 1A (*n* = 12, see Table 2). *SOZ* seizure onset zone, *SIS* stimulation-induced seizures, *LVFA* low voltage fast activity.

Neither the SIS-inducing electrodes nor the SOZ of the SIS were fully concordant with the SOZ of the spontaneous seizures (Fig. 3). As mentioned above, GLMM were used to investigate potential SIS predictors of the spontaneous SOZ. Table 2 summarizes the analyses in the total cohort and in the two complementing subcohorts defined by the postoperative Engel criterion. In the subgroup of patients with the postoperative Engel outcome of 1A, the ROC curves provided an AUC for the correct prediction of the spontaneous SOZ of 0.876 for the SOZ of SIS. In this subgroup the AUC for the prediction parameters for the localization of the spontaneous SOZ were “SOZ of induced seizures” > “LVFA at onset of SIS” > “identical onset pattern of SIS” > “SIS inducing electrodes” > “identical semiology of SIS” > “atypical semiology of SIS” > “untypical onset pattern of SIS”, Fig. 4. In the sub-cohort with poorer postoperative outcome (Engel ≥ 1B), the AUC values were much lower.

**Table 2:** Areas under the receiver operating characteristic curves based on the predicted probabilities post-estimated from the models.

Predictors of spontaneous SOZ	Total cohort (*n* = 20)		Engel 1A (*n* = 12)		Engel ≥ 1B (*n* = 8)	
	AUC	SE [CI 95%]	AUC	SE [CI 95%]	AUC	SE [CI 95%]
SOZ of SIS	0.806	0.022 [0.763–0.849]	0.876	0.021 [0.834–0.917]	0.684	0.045 [0.596–0.773]
LVFA at onset of SIS	0.801	0.021 [0.761–0.842]	0.860	0.022 [0.817–0.902]	0.638	0.044 [0.552–0.724]
Identical onset pattern of SIS	0.802	0.021 [0.760–0.844]	0.858	0.025 [0.810–0.906]	0.612	0.046 [0.523–0.701]
SIS inducing electrodes	0.773	0.022 [0.730–0.817]	0.812	0.024 [0.766–0.858]	0.702	0.045 [0.614–0.789]
Identical semiology of SIS	0.777	0.021 [0.735–0.818]	0.804	0.023 [0.758–0.849]	0.690	0.046 [0.601–0.780]
Untypical semiology of SIS	0.710	0.027 [0.656–0.763]	0.780	0.032 [0.717–0.843]	0.674	0.041 [0.593–0.754]
Untypical onset pattern of SIS	0.726	0.024 [0.680–0.773]	0.757	0.029 [0.700–0.813]	0.685	0.042 [0.603–0.767]

Values are *AUC* area under the curve, *SE* standard error, *CI 95%* confidence interval, *n* number, *SOZ* seizure onset zone, *SIS* stimulation-induced seizures, *LVFA* low voltage fast activity, Engel postsurgical outcome scale.

In the next step we analyzed the odds to observe the spontaneous SOZ in a model, with the five explanatory variables with the largest AUCs and the confounders. The model revealed Intra-Cluster Correlation Coefficient (ICC) of 0.112, which points at a rather small level of within-patient homogeneity; nevertheless, the mixed-effect model fits the data significantly better than the naïve logistic model did (Likelihood Ratio Test, data not shown). Assuming that the model was correctly specified, and holding all other variables in the model constant, the odds of observing spontaneous SOZ in a patient with a SIS who had “SIS inducing electrodes” and “LVFA at onset of SIS” were 55 times larger than the odds for the same patient without SIS inducing electrodes and without LVFA at onset of SIS, Table 3. LVFA as seizure onset pattern of the electrically induced seizure had a larger probability of predicting the spontaneous SOZ than the outcome that did not include this variable.

**Table 3:** SOZ prediction by combination of SIS variables.

SOZ prediction by combination of variables	OR	SE	CI
“SIS inducing electrodes” + “LVFA at onset of SIS”	54.620	40.153	[12.930–230.727]
“Identical onset pattern of SIS” + “LVFA at onset of SIS”	38.095	21.389	[12.675–114.498]
“SOZ of SIS” + “LVFA at onset of SIS”	24.053	15.714	[6.685–86.547]
“Identical semiology of SIS” + “LVFA at onset of SIS”	15.805	10.387	[4.359–57.309]
“SIS inducing electrodes” **+** “identical onset pattern of SIS”	12.267	7.779	[3.539–42.515]
“SOZ of SIS” **+** “SIS inducing electrodes”	7.745	3.213	[3.435–17.464]
“SIS inducing electrodes” + “identical semiology of SIS”	5.089	2.786	[1.740–14.882]
“Identical onset pattern of SIS” + “identical semiology of SIS”	3.550	2.262	[1.018–12.373]

Estimated odd ratios of the outcome SOZ based on the full GLMM model with the variables of interests (details presented in the Supplement) Values are *OR* odds ratio, *SE* standard error, *CI 95%* confidence interval, *SOZ* seizure onset zone, *SIS* stimulation-induced seizures, *LVFA* low voltage fast activity.

## Discussion

### Main findings of our study

Our results show that stimulation-induced seizures (SIS) provide added clinical value beyond spontaneous seizures.

First, our results describe characteristics of SIS that are predictors of a good postoperative outcome in case of resection. These comprise SIS with the typical seizure pattern, electrodes involved in the seizure pattern, seizure inducing electrodes, low voltage fast activity (LVFA) at SIS onset and SIS with the typical semiology. In particular, a high percentage of resected electrode contacts with LVFA (80%) was seen in patients with a good outcome.

Secondly, our results describe the characteristics of SIS that best predict the epileptogenic zone. The best predictors of the spontaneous SOZ in our statistical analysis were the SIS onset zone, LVFA at SIS seizure onset and an identical seizure pattern of SIS. We also analyzed the predictive value of the most relevant combinations of two predictors. We found an outstandingly good prediction of the spontaneous SOZ for combinations including LVFA at SIS onset.

### Stimulation-induced seizure onset zone and seizure inducing electrode contacts

Our data show an association between the percentage of resected seizure-inducing electrodes and postoperative outcome, as also shown by Oderiz et al.^12^In addition, we showed that SIS electrodes are predictors of the spontaneous SOZ. Since we have documented in our EEG recordings that seizure patterns do not necessarily start at stimulated electrodes, we also analyzed both features separately. The concept of SIS is based on the assumption that stimulation of any area functionally connected to the epileptogenic zone can induce seizures^5,24^. The predictors identified in this study are intended to help discriminate between stimulation induced seizures in the epileptogenic zone and SIS in areas outside the spontaneous SOZ. We documented LVFA at onset both in the area of the stimulated electrode and in more distant electrodes. Electrodes whose stimulation induce seizures with LVFA at onset represent an important epileptogenic network node and therefore should be resected^25^.

### Typical seizure pattern and LVFA in stimulation-induced seizures as a predictor of the epileptogenic zone

Previous studies have shown that spontaneous LVFA is a pattern that is associated with a high degree of epileptogenicity^26^. Brain areas where electroclinical seizures can be induced are a more reliable marker of epileptogenicity than after-discharges as an EEG phenomenon without clinical correlate^7,13^. In previous studies, LVFA was described as the most common seizure pattern in spontaneous seizures and was observed in various pathologies^27^. The presence of LVFA in spontaneous seizures is significantly associated with a better post-operative outcome^14^. Consistent with these findings, our results show that the occurrence of LVFA is also a relevant prognostic feature during SIS. There was a high share of resected electrode contacts with stimulated LVFA (80%) in patients with a good postoperative outcome. Furthermore, stimulated LVFA was an excellent predictor of the SOZ with an AUC of 0.860. In combination with other predictors, the highest predictive values for the spontaneous SOZ were seen when LVFA was added. From our results, GLMM models that included LVFA were more likely to predict the spontaneous SOZ than the models that did not include this variable. This conclusion was only possible because all predictors in all models were binary, i.e. measured on the same scale.

### Semiology in stimulation-induced seizures (SIS) as a predictor of the epileptogenic zone

The concordance of semiology and site of seizure origin between spontaneous and provoked seizures is reported to be around 70–90%^7,9,28,29^. Stimulation seizures, in which the electrically induced seizure manifestations are identical to those of spontaneous seizures, have been described as a good prognostic determinant^12,11^. We could confirm these data, but semiology or combinations of predictors including semiology were inferior to LVFA in respect of predicting postoperative seizure freedom or localization of spontaneous SOZ in our study. Because spontaneous seizures sometimes may arise in non-eloquent areas of the cortex and the first symptom of habitual seizures may occur during the propagation of ictal activity, stimulation-induced symptomatology matching the typical semiology may incorrectly localize the SOZ.

Seizure semiology is influenced by the stimulation frequency used. A study by Sivaraju et al. reports that SIS were less frequent overall with a stimulation frequency of 1 Hz, over 80% of these were habitual seizures^11^. However, SIS with 1 Hz stimulation occurred in more insular and medial temporal stimulation sites. In contrast, SIS with 50 Hz stimulation were more frequent, but with a rate of 35% atypical seizures^11^. Therefore, the predictors shown in our study may not easily be transferred to other stimulation frequencies.

### Benefits of stimulation-induced seizures (SIS) in the clinical setting

Stimulation-induced seizures are valuable in preoperative evaluation. In patients in whom a spontaneous seizure cannot be recorded during invasive monitoring, SIS may provide additional information about the SOZ. In patients in whom the SOZ cannot be delineated with certainty due to recording of a spontaneous seizure, SIS may add diagnostic details about the epileptogenic network. Moreover, SIS may provide a patient-focused opportunity to reduce the duration of invasive monitoring by time-efficient recording of SIS. Future studies should investigate whether SIS can replace spontaneous seizures. Furthermore, the analysis of SIS may help to plan the electrode contacts for radiofrequency thermocoagulation or laser ablation^30^.

### 50 Hz electrical stimulation for seizure induction

In our center, the use of high-frequency stimulation at 50 Hz has become established. However, some centers use low-frequency stimulation at 1 Hz. The characteristics of both stimulation frequencies have been studied in the literature^12,11,28,29^. On the one hand, high-frequency stimulation has been associated with higher sensitivity in inducing seizures at lower specificity, often inducing false-positive seizures^11^. On the other hand, low-frequency stimulation has been associated with a low likelihood of inducing false-positive seizures, although it may be less efficient at provoking seizures^11^. The risk of convulsive seizures may be lower with low-frequency stimulation^11^. Low-frequency stimulation of medial temporal or insular regions was more likely to induce seizures than stimulation of other brain regions^11^. Due to the different characteristics of high and low-frequency stimulation, future studies should also investigate the stimulation frequencies separately.

### Strength and limitations

The strength of our study is the detailed analysis of a cohort receiving intracranial electrical stimulation and epilepsy surgery with a 1-year follow-up. We used a standardized stimulation protocol with systematic bipolar stimulation at 50 Hz. In addition, we could perform a detailed electroclinical analysis of semiology, seizure pattern and involvement of electrode contacts in the seizure pattern and were able to precisely correlate electrode contacts with the anatomy and with the resection zone. This detailed electroclinical analysis of our data allowed us to confirm existing and add new predictors of SOZ and postoperative outcome for SIS and, in particular, to describe the high predictive power for the occurrence of LVFA.

Nevertheless, our study suffered from several limitations, most notably the retrospective design and moderate sample size, which may overestimate the effect size and limit generalizability. Concerning the GLMM analysis it needs to be emphasized that a direct comparison of the odds ratios is valid only because in our experimental setting all relevant predictors are binary, and hence measured on the same scale. Interpretation of the effects of these characteristics is identical (effect of the change from “absent” to “present”). It also needs to be noted that the GLMM paradigm leads to a conditional (= a patient-specific) inference; as an alternative, one might consider Generalized Estimating Equations (GEE) methodology to obtain population (= marginal) interpretation.

Due to the study design, our study can say little about the potential additional benefit of removing electrode contacts identified by SIS beyond the spontaneous SOZ in cases where there is no overlap between the spontaneous and stimulation SOZ. Future prospective and multicenter studies should investigate the prognostic characteristics of SIS.

## Conclusions

Stimulation-induced seizures are an additional aid for the clinician to delineate the area to be removed in epilepsy surgery. Characteristics such as resection of the seizure onset zone, the seizure pattern and the semiology of the stimulation-induced seizure predict seizure freedom. Electrodes should be resected where stimulation-induced seizures have been generated or the stimulation seizure pattern was localized at onset if low-voltage fast activity occurred. Future studies should evaluate the prognostic characteristics of stimulation-induced seizures for different stimulation frequencies in a larger cohort of patients.

## Electronic supplementary material

Below is the link to the electronic supplementary material.

Supplementary Material 1

Supplementary Material 2

## Data Availability

The datasets used and/or analysed during the current study are available from the corresponding author on reasonable request.

## Abbreviations

CI: Confidence interval
EEG: Electroencephalography
IQR: Interquartile range
OR: Odds ratio
SD: Standard deviation
SIS: Stimulation-induced seizures
SOZ: Spontaneous seizure onset zone
LVFA: Low voltage fast activity
ROC: Receiver operating characteristic
AUC: Area under the curve

## References

[1] Wiebe, S., Blume, W. T., Girvin, J. P. & Eliasziw, M. A randomized, controlled trial of surgery for temporal-lobe epilepsy. *N. Engl. J. Med.* **345** (5), 311–318 (2001). 11484687 10.1056/NEJM200108023450501

[2] Engel, J. et al. Early surgical therapy for drug-resistant temporal lobe epilepsy: A randomized trial. *Jama* **307** (9), 922–930 (2012). 22396514 10.1001/jama.2012.220 PMC4821633

[3] Jehi, L. et al. Development and validation of nomograms to provide individualised predictions of seizure outcomes after epilepsy surgery: A retrospective analysis. *Lancet Neurol.* **14** (3), 283–290. 10.1016/S1474-4422(14)70325-4 (2015). 25638640 10.1016/S1474-4422(14)70325-4

[4] Rosenow, F. & Lüders, H. Presurgical evaluation of epilepsy patients. *Medicina* **44** (8), 585–592 (2008). 18791335

[5] David, O., Bastin, J., Chabardès, S., Minotti, L. & Kahane, P. Studying network mechanisms using intracranial stimulation in epileptic patients. *Front. Syst. Neurosci.* **4**, 1–10 (2010). 21060722 10.3389/fnsys.2010.00148 PMC2972750

[6] George, D. D., Ojemann, S. G., Drees, C. & Thompson, J. A. Stimulation mapping using stereoelectroencephalography: current and future directions. *Front. Neurol.* **11**, 1–7 (2020). 32477236 10.3389/fneur.2020.00320 PMC7238877

[7] Bernier, G. P. et al. Electrical stimulation of the human brain in epilepsy. *Epilepsia* **31** (5), 513–520 (1990). 2401243 10.1111/j.1528-1157.1990.tb06099.x

[8] Wieser, H. G., Bancaud, J., Talairach, J., Bonis, A. & Szikla, G. Comparative value of spontaneous and chemically and electrically induced seizures in establishing the lateralization of temporal lobe seizures. *Epilepsia* **20** (1), 47–59 (1979). 421676 10.1111/j.1528-1157.1979.tb04775.x

[9] Schulz, R. et al. Localization of epileptic auras induced on stimulation by subdural electrodes. *Epilepsia* **38** (12), 1321–1329 (1997). 9578528 10.1111/j.1528-1157.1997.tb00070.x

[10] Chassoux, F. et al. Stereoelectroencephalography in focal cortical dysplasia. A 3D approach to delineating the dysplastic cortex. *Brain* **123** (8), 1733–1751 (2000). 10908202 10.1093/brain/123.8.1733

[11] Sivaraju, A. et al. Systematic 1 Hz direct electrical stimulation for seizure induction: A reliable method for localizing seizure onset zone and predicting seizure freedom. *Brain Stimul.* **17** (2), 339–345. 10.1016/j.brs.2024.03.011 (2024). 38490472 10.1016/j.brs.2024.03.011

[12] Cuello Oderiz, C. et al. Association of cortical stimulation-induced seizure with surgical outcome in patients with focal drug-resistant epilepsy. *JAMA Neurol.* **76** (9), 1070–1078 (2019). 31180505 10.1001/jamaneurol.2019.1464 PMC6563597

[13] Gollwitzer, S. et al. Afterdischarges elicited by cortical electric stimulation in humans: when do they occur and what do they mean? *Epilepsy Behav.* **87**, 173–179. 10.1016/j.yebeh.2018.09.007 (2018). 30269940 10.1016/j.yebeh.2018.09.007

[14] Lagarde, S. et al. The repertoire of seizure onset patterns in human focal epilepsies: determinants and prognostic values. *Epilepsia* **60** (1), 85–95 (2019). 30426477 10.1111/epi.14604

[15] Reindl, C. et al. Resection of dominant fusiform gyrus is associated with decline of naming function when Temporal lobe epilepsy manifests after the age of five: A voxel-based lesion-symptom mapping study. *NeuroImage Clin.* **35**, 1–2 (2022). 10.1016/j.nicl.2022.103129 PMC9421498 36002957

[16] Reindl, C. et al. Age of epilepsy onset as modulating factor for naming deficit after epilepsy surgery: a voxel-based lesion-symptom mapping study. *Sci. Rep.* **13** (1), 1–8. 10.1038/s41598-023-40722-4 (2023). 37658152 10.1038/s41598-023-40722-4 PMC10474263

[17] Rorden, C. & Brett, M. *Stereotaxic Display of Brain Lesions*. www.fil.ion.ucl.ac.uk/spm/ (2022). 10.1155/2000/421719 11568431

[18] Nachev, P., Coulthard, E., Jäger, H. R., Kennard, C. & Husain, M. Enantiomorphic normalization of focally lesioned brains. *Neuroimage* **39** (3), 1215–1226 (2008). 18023365 10.1016/j.neuroimage.2007.10.002 PMC2658465

[19] Ashburner, J. & Friston, K. J. Unified segmentation. *Neuroimage* **26** (3), 839–851 (2005). 15955494 10.1016/j.neuroimage.2005.02.018

[20] Jenkinson, M., Beckmann, C. F., Behrens, T. E. J., Woolrich, M. W. & Smith, S. M. FSL. *Neuroimage* **62** (2), 782–790 (2012). 21979382 10.1016/j.neuroimage.2011.09.015

[21] *2011 Brainstorm—A User-Friendly Application for MEG EEG Analysis.pdf*. 10.1155/2011/879716 PMC3090754 21584256

[22] Team, R. C. *A Language and Environment for Statistical Computing* (R Foundation Statistics Computing, 2018).

[23] Cleves, M. A. From the help desk: comparing areas under receiver operating characteristic curves from two or more probit or logit models. *Stata J. Promot Commun. Stat. Stata* **2** (3), 301–313 (2002).

[24] Trébuchon, A. & Chauvel, P. Electrical stimulation for seizure induction and functional mapping in stereoelectroencephalography. *J. Clin. Neurophysiol.* **33** (6), 511–521 (2016). 27918346 10.1097/WNP.0000000000000313

[25] Gupta, K., Grover, P. & Abel, T. J. Current conceptual Understanding of the epileptogenic network from stereoelectroencephalography-based connectivity inferences. *Front. Neurol.* **11**, 1–7 (2020). 33324320 10.3389/fneur.2020.569699 PMC7724044

[26] Bartolomei, F., Chauvel, P. & Wendling, F. Epileptogenicity of brain structures in human Temporal lobe epilepsy: A quantified study from intracerebral EEG. *Brain* **131** (7), 1818–1830 (2008). 18556663 10.1093/brain/awn111

[27] Perucca, P., Dubeau, F. & Gotman, J. Intracranial electroencephalographic seizure-onset patterns: effect of underlying pathology. *Brain* **137** (1), 183–196 (2014). 24176980 10.1093/brain/awt299

[28] Spilioti, M. et al. The nature, frequency and value of stimulation induced seizures during extraoperative cortical stimulation for functional mapping. *Seizure* **81**, 71–75. 10.1016/j.seizure.2020.07.027 (2020). 32763786 10.1016/j.seizure.2020.07.027

[29] Kovac, S., Kahane, P. & Diehl, B. Seizures induced by direct electrical cortical stimulation—Mechanisms and clinical considerations. *Clin. Neurophysiol.* **127** (1), 31–39. 10.1016/j.clinph.2014.12.009 (2016). 25613034 10.1016/j.clinph.2014.12.009

[30] Dimova P, de Palma L, Job-Chapron AS et al. Radiofrequency thermocoagulation of the seizure-onset zone during stereoelectroencephalography. *Epilepsia ***58**(3), 381-392. 10.1111/epi.13663 (2017). Epub 2017 Feb 2. PMID: 28150296. 10.1111/epi.13663 28150296

