# Supplementary figures and images for: The prognostic value of cortical stimulation induced seizures using stereo EEG in presurgical evaluation of focal epilepsies
Source: Sci Rep. 2025 Mar 7;15:7941. doi: 10.1038/s41598-025-92241-z (PMC11885602; doi:10.1038/s41598-025-92241-z)

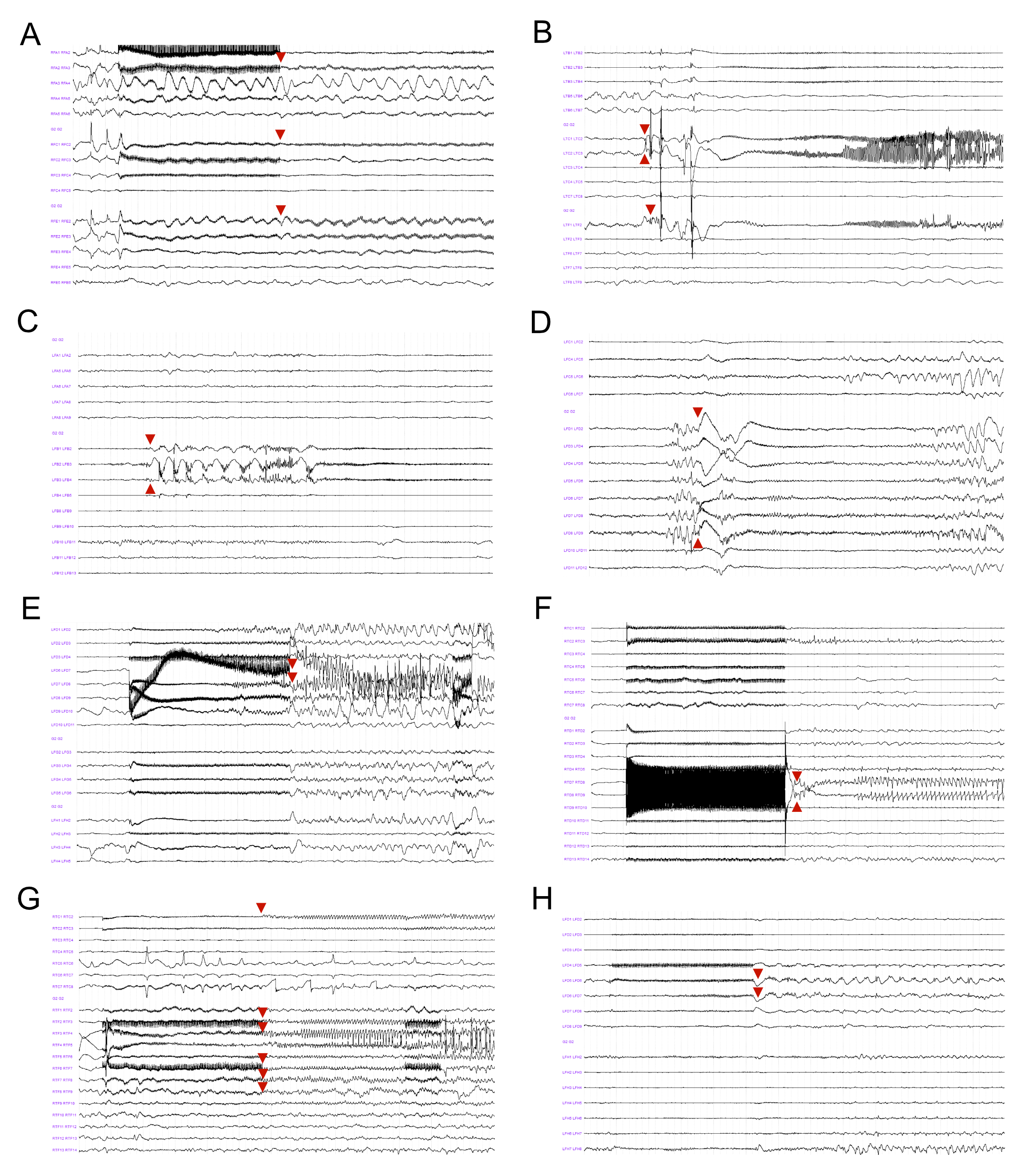

Supplement: Supplementary file 1 — Supplementary Material 1 [file 41598_2025_92241_MOESM1_ESM.tiff]
