# Supplementary material for: The prognostic value of cortical stimulation induced seizures using stereo EEG in presurgical evaluation of focal epilepsies
Source: Sci Rep. 2025 Mar 7;15:7941. doi: 10.1038/s41598-025-92241-z (PMC11885602; doi:10.1038/s41598-025-92241-z)
Supplement: Supplementary file 2 — Supplementary Material 2 [file 41598_2025_92241_MOESM2_ESM.pdf]

Supplementary Information

Table S1: Fixed effects in the model with the outcome variable SOZ

	**Coefficient**	**SE**	**CI**	**z**	**p-value**
SOZ of SIS	.613	.462	[-.293 - 1.520]	1.33	0.185
Identical onset pattern of SIS	1.073	.467	[.158 - 1.990]	2.30	**0.022**
Identical semiology of SIS	.194	.431	[-.651 - 1.038]	0.45	0.653
SIS inducing electrodes	1.434	.374	[.701 - 2.166]	3.83	**< 0.001**
LVFA at onset of SIS	2.567	.551	[1.486 - 3.647]	4.66	**< 0.001**
Engel outcome 1	-.513	.482	[-1.457 - .431]	-1.06	0.287
Lesional epilepsy	-.614	.536	[-1.664 - .435]	-1.15	0.251
Temporal lobe epilepsy	-.181	1.074	[-2.286 - 1.924]	-0.17	0.866
Extratemporal epilepsy	-.744	1.165	[-3.027 - 1.540]	-0.64	0.523
Left hemispheric epilepsy	-.117	.581	[-1.256 - 1.022]	-0.20	0.841
Epilepsy onset (years)	-.021	.581	[-.096 - .054]	-0.55	0.584
Duration of epilepsy (years)	-.029	.0263	[-.080 - .023]	-1.10	0.272
Female	.590	.465	[-.321 - 1.501]	1.27	0.204

Values are SE standard error, CI 95% confidence interval, SOZ seizure onset zone, SIS stimulation-induced seizures, LVFA low voltage fast activity

Supplementary figure legend

Figure S1: Classification of stimulation seizure patterns according to Lagarde et al. into 8 different patterns (A: LVFA, B: preictal spiking followed by LVFA, C: burst of polyspikes followed by LVFA, D: slow wave or baseline shift followed by LVFA, E: rhythmic spikes or spike-waves, F: sharp theta/alpha activity, G: sharp beta activity, H: delta and fast activity). Invasive EEG is depicted with High-pass filter: 0.3Hz, Low-pass filter: 1000Hz, Gain: 800µV/cm, Notch-filter on. [A, E, F, G and H depict stimulation-induced seizures; B, C and D show spontaneous seizures.]
